# BET-inhibitors as sensitizers for BH3-mimetics

**DOI:** 10.18632/aging.101259

**Published:** 2017-06-26

**Authors:** Chiaki Tsuge Ishida, Georg Karpel-Massler, Markus D. Siegelin

**Affiliations:** Department of Clinical and Biological Sciences, University of Turin, 10043 Turin, Italy

**Keywords:** Bromodomain Extraterminal domain proteins inhibitors, BH3-mimetics, drug combination therapies, cell death, cancer

The transcription factor c-myc is known to be an important molecule in a couple of malignancies, most notably in Burkitt-lymphoma, but also in other cancers. However, malignant brain tumors were also shown to be dependent on c-myc, especially brain tumor initiating cells (BIC) [1]. BIC were identified more than a decade ago and it was believed that this fraction of tumor cells is at least in part responsible for the highly aggressive behavior of glioblastomas [2], such as the development of radiation resistance. Along with other stem cell markers, e.g. Nestin, CD133, Musashi, BIC express high levels of c-myc and knockdown of c-myc impacts their viability and tumor xenografts derived from BIC with silenced c-myc display attenuated growth. Since c-myc is a transcription factor, this protein was considered to be “undruggable”. With the recent discovery that Bromodomain Extraterminal domain proteins (BET), such as BRD4, control the levels of c-myc it became evident that interference with BRD proteins will affect c-myc activity [3]. While BRD proteins controls the transcription of c-myc, it more broadly also affects RNA-polymerase-II [4]. Utilizing high-throughput drug screening, a compound family of BET-inhibitors was identified. The most prominent molecule out of this family is the thienotriazolodiazepine, JQ1. Through chemical modification of the core structure of JQ1 several additional inhibitors were derived, such as OTX015 and others. The JQ1 derivatives display more favorable pharmacokinetic properties, easing administration to patients. JQ1 potently inhibited the expression of c-myc both at the transcriptional and protein level. Consistently, cell lines and xenografts with high levels of c-myc displayed a high susceptibility to JQ1. Our recent work suggests that JQ1 is active against glioblastoma cell lines and especially against stem cell-like glioma or brain tumor initiating cells. While there are initial impressive responses to these BRD inhibitors, resistance to therapy inevitably emerges. Therefore, combination therapies, involving these novel c-myc inhibitors are warranted. Established c-myc downstream targets involve the anti-apoptotic Bcl-2 family member, Bcl-xL, amongst others, suggesting that interference with c-myc or BRD4 might sensitize for intrinsic apoptosis. Recently, we found that BET-inhibitors, JQ1 and OTX015, broadly enhance the cell death inducing effects of BH3-mimetics [5], such as ABT263 and Obatoclax [6]. The combination treatment dramatically facilitated apoptosis as illustrated by enhanced staining with Annexin V and caspase cleavage. JQ1 treatment had an impact on the expression of pro- and anti-apoptotic molecules, suggesting that these modulations might be implicated in the enhanced cell death mediated by the combination treatment. JQ1 displayed a remarkable increase in BIM protein levels in all model systems tested. Moreover, the BET-inhibitors elicited an effect on Mcl-1 levels, which is one of the most relevant mediators of resistance towards BH3-mimetic mediated apoptosis. In addition, the combination treatment of ABT263 and JQ1 affected the levels of the protein Noxa, which is encoded by the *PMAIP1* gene. Previous research has shown that Noxa is an important mediator for the sensitivity of cancer cells to BH3-mimetics. Our finding that Noxa knockdown protects from ABT263/JQ1 mediated cell death supports this idea. The deeper question as to how the combination treatment increases Noxa opens up one particular possibility, which is the activation of an integrated stress response. Our findings suggest that the Noxa increase depends on the transcription factor, ATF4. Regulation of ATF4 occurs through the integrated stress response. For instance, the endoplasmic reticulum kinase, PERK, phosphorylates eif2α, driving selective up regulation of certain stress related proteins, harboring an upstream open reading frame uORF, including ATF4. However, other kinases, such as PKR or GCN2, may activate selective protein via ATF4 synthesis as well. How ATF4 is up regulated by the combination treatment of BH3-mimetics and BET-inhibitors remains to be elucidated. However, since JQ1 via c-myc has a vital impact on energy metabolism, it may be plausible to consider that loss of ATP might mediate a stress response, starting in the endoplasmic reticulum with activation of PERK and ATF4. Our findings suggested in vivo activity of ABT263 and the JQ1 derivative, OTX015. However, additional more detailed studies, for instance in orthotopic xenografts, are necessary to judge the overall impact. Our findings are supported by other groups, showing similar findings in other tumor entities, involving the selective Bcl-2 inhibitor, ABT199 [7, 8] which recently was designated for accelerated FDA approval. All in all, the combination treatment of BH3-mimetics and BET-inhibitors appears to be a potential treatment strategy for several malignancies and should be explored further.
